# Supplementary material for: Taperin bundles F-actin at stereocilia pivot points enabling optimal lifelong mechanosensitivity
Source: J Cell Biol. 2025 Jun 5;224(8):e202408026. doi: 10.1083/jcb.202408026 (PMC12139522; doi:10.1083/jcb.202408026)
Supplement: Table S1 — shows the reported pathogenic or likely pathogenic variants of human TPRN and associated phenotypes. [file jcb_202408026_tables1.docx]

Table S1. **Reported pathogenic or likely pathogenic variants of human *TPRN* and associated phenotypes.**

| cDNA | Protein | Reported phenotype | PMID/URL |
| --- | --- | --- | --- |
| c.53G>A | Trp18* | Sensorineural hearing loss | 31541171 |
| c.199G>C | Glu67Gln | Hearing loss | 26969326 |
| c.559G>T | Ala187Ser | Non-syndromic hearing loss | 25129962, 30872718 |
| c.1038G>A | Glu346Glu | Autism | 35982159 |
| c.1159G>T | Glu387* | Sensorineural hearing loss | 31541171 |
| c.1239G>A | Trp413* | Non-syndromic hearing loss | 20170899 |
| c.1326C>G | Ala442Ala | Orofacial clefting | 32574564 |
| c.1486C>T | Arg496Trp | Developmental disorder | 35982159 |
| c.1513C>T | Pro505Ser | Hearing loss | 27610647 |
| c.1758G>A | Gln586Gln | Autism spectrum disorder | 35982160, 35982159 |
| c.1927G>A | Val643Met | Microtia | 28968992 |
| c.1725+5G>A |  | Hearing loss | 26969326 |
| c.2073+8G>T |  | Hearing loss | 36597107 |
| c.1964dup | p.(Leu4Argfs*9)^#^ | Hearing loss | https://deafnessvariationdatabase.org/gene/TPRN |
| c.117delC | p.(Ala41Argfs*409) | Hearing loss | 38844983 |
| c.225_235del11 | p.(Gly76Alafs*150) | Non-syndromic hearing loss | 20170898, 31980526, 23340767 |
| c.650delG | p.(Gly217Alafs*233) | Non-syndromic hearing loss | 34599366 |
| c.789delC | p.(Ser264Alafs*186) | Non-syndromic hearing loss | 34599366 |
| c.1427delC | p.(Pro476Argfs*67) | Non-syndromic hearing loss | 20170899 |
| c.1530delG | p.(Thr511Leufs*32) | Non-syndromic hearing loss | 20170898 |
| c.225_235dup11 | p.(Leu79Argfs*375) | Hearing loss | 38374194 |
| c.227_237dup11 | p.(Leu80Glyfs*374) | Non-syndromic hearing loss | 20170899 |
| c.440_444dupGCCGC | p.(Arg149Alafs*303) | Hearing loss | 32279305 |
| c.701_705dupCTGCC | p.(Asn236Leufs*216) | Non-syndromic hearing loss | 26226137 |
| c.732dupG | p.(Ser245Valfs*38) | Non-syndromic hearing loss | 37217689 |
| c.788_789dupCC | p.(Ser264Profs*187) | Non-syndromic hearing loss | 34599366 |
| c.943dupC | p.(Leu315Profs*32) | Hearing loss | 26969326 |
| c.1836_1844dupGGAGGAGGA | p.(Glu619_Glu621dup) | Hearing loss | 36597107 |
| c.1964dup | p.(Gly656Argfs*44)^#^ | Hearing loss | https://deafnessvariationdatabase.org/gene/TPRN |

^#^Two variants were identified as likely pathogenic in DVD; *stop codon; updated as of 2/10/2025

These variants evaluated using the Human Gene Mutation Database (HGMD) (https://www.hgmd.cf.ac.uk/ac/index.php), Simple ClinVar (http://simple-clinvar.broadinstitute.org) and Deafness Variation Database (<http://otoscope.eng.uiowa.edu/dvd>)
