# Supplementary material for: Taperin bundles F-actin at stereocilia pivot points enabling optimal lifelong mechanosensitivity
Source: J Cell Biol. 2025 Jun 5;224(8):e202408026. doi: 10.1083/jcb.202408026 (PMC12139522; doi:10.1083/jcb.202408026)
Supplement: Table S2 — shows the statistical analysis of ABR data for Tprn−/−, Tprn+/−, and Tprn+/+ mice. [file jcb_202408026_tables2.docx]

Table S2. **Statistical analysis of ABR data for *Tprn^-/-^*, *Tprn^+/-^*, and *Tprn^+/+^* mice.**

|  | **Estimate** | **95% CI** | ***s.e.*** | ***t* value** | ***p value*** |
| --- | --- | --- | --- | --- | --- |
| (Intercept) | 14.31 | [8.06, 20.54] | 3.27 | 4.38 | 2.8E-05*** |
| Genotype *Tprn^+/+^* | Reference |  |  |  |  |
| *Tprn^+/-^* | 3.03 | [-3.90, 9.96] | 3.63 | 0.83 | 0.41 |
| *Tprn^-/-^* | 24.32 | [16.77, 31.93] | 3.96 | 6.14 | 2.1E-08*** |
| Age P18 | Reference |  |  |  |  |
| P30 | 3.80 | [-6.05, 13.90] | 5.17 | 0.74 | 0.46 |
| P60 | 5.24 | [-1.91, 12.39] | 3.69 | 1.42 | 0.16 |
| Frequency 8 kHz | Reference |  |  |  |  |
| 16 kHz | 4.95 | [1.53, 8.36] | 1.76 | 2.81 | 5.4E-03** |
| 32 kHz | 16.52 | [13.10, 19.94] | 1.76 | 9.38 | 8.7E-18*** |
| Genotype x Age *Tprn^+/+^* P18 | Reference |  |  |  |  |
| *Tprn^+/-^* P30 | 2.24 | [-9.83, 14.40] | 6.31 | 0.36 | 0.72 |
| *Tprn^-/-^* P30 | 28.47 | [16.41, 40.12] | 6.12 | 4.66 | 5.3E-06*** |
| *Tprn^+/-^* P60 | 7.06 | [-1.35, 15.46] | 4.33 | 1.63 | 0.10 |
| *Tprn^-/-^* P60 | 44.46 | [35.31, 53.61] | 4.72 | 9.43 | 6.3E-18*** |
| Observations: | 276 |  |  |  |  |
| Subjects: | 46 |  |  |  |  |
