# Supplementary material for: Taperin bundles F-actin at stereocilia pivot points enabling optimal lifelong mechanosensitivity
Source: J Cell Biol. 2025 Jun 5;224(8):e202408026. doi: 10.1083/jcb.202408026 (PMC12139522; doi:10.1083/jcb.202408026)
Supplement: Table S3 — shows the comparisons between time points of Tprn−/−, Tprn+/−, and Tprn+/+ ABR data within genotypes. [file jcb_202408026_tables3.docx]

Table S3. **Comparisons between time points of *Tprn^-/-^*, *Tprn^+/-^*, and *Tprn^+/+^* ABR data within genotypes.**

| ***Tprn^+/+^*** | **Estimate** | ***s.e.*** | ***df*** | ***t* value** | ***p* value** |
| --- | --- | --- | --- | --- | --- |
| P30 - P18 | 3.80 | 5.21 | 251.34 | 0.73 | 0.75 |
| P60 - P18 | 5.24 | 3.69 | 224.22 | 1.42 | 0.33 |
| P60 - P30 | 1.43 | 5.21 | 251.34 | 0.28 | 0.96 |
| ***Tprn^+/-^*** |  |  |  |  |  |
| P30 - P18 | 6.05 | 3.64 | 207.27 | 1.66 | 0.22 |
| P60 - P18 | 12.30 | 2.28 | 229.59 | 5.40 | 5.1E-07*** |
| P60 - P30 | 6.25 | 3.66 | 209.58 | 1.71 | 0.21 |
| ***Tprn^-/-^*** |  |  |  |  |  |
| P30 - P18 | 32.28 | 3.29 | 247.93 | 9.82 | 1.1E-13*** |
| P60 - P18 | 49.70 | 2.94 | 224.22 | 16.90 | 1.6E-15*** |
| P60 - P30 | 17.42 | 3.29 | 247.93 | 5.30 | 7.7E-07*** |
