# Supplementary material for: Taperin bundles F-actin at stereocilia pivot points enabling optimal lifelong mechanosensitivity
Source: J Cell Biol. 2025 Jun 5;224(8):e202408026. doi: 10.1083/jcb.202408026 (PMC12139522; doi:10.1083/jcb.202408026)
Supplement: Table S4 — shows the statistical analysis of Tprn−/−, Tprn+/−, and Tprn+/+ ABR data at each frequency. [file jcb_202408026_tables4.docx]

Table S4. **Statistical analysis of *Tprn^-/-^*, *Tprn^+/-^*, and *Tprn^+/+^* ABR data at each frequency.**

| **8 kHz** | **Estimate** | **95% CI** | ***s.e.*** | ***t* value** | ***p value*** |
| --- | --- | --- | --- | --- | --- |
| (Intercept) | 19.29 | [11.00, 27.57] | 4.40 | 4.38 | 3.4E-05*** |
| Genotype *Tprn^+/+^* | Reference |  |  |  |  |
| *Tprn^+/-^* | 0.98 | [-8.71, 10.67] | 5.15 | 0.19 | 0.85 |
| *Tprn^-/-^* | 14.81 | [4.21, 25.40] | 5.63 | 2.63 | 0.01* |
| Age P18 | Reference |  |  |  |  |
| P30 | 2.38 | [-12.74, 17.50] | 8.04 | 0.30 | 0.77 |
| P60 | -1.43 | [-13.14, 10.29] | 6.23 | -0.23 | 0.82 |
| Genotype x Age *Tprn^+/+^* P18 | Reference |  |  |  |  |
| *Tprn^+/-^* P30 | 7.36 | [-10.92, 25.63] | 9.72 | 0.76 | 0.45 |
| *Tprn^-/-^* P30 | 27.53 | [9.63, 45.43] | 9.52 | 2.89 | 4.9E-03** |
| *Tprn^+/-^* P60 | 8.39 | [-5.37, 22.14] | 7.31 | 1.15 | 0.25 |
| *Tprn^-/-^* P60 | 59.61 | [44.62, 74.60] | 7.97 | 7.48 | 6.9E-11*** |
|  |  |  |  |  |  |
| **16 kHz** |  |  |  |  |  |
| (Intercept) | 21.55 | [14.01, 29.11] | 4.01 | 5.38 | 7.0E-07*** |
| Genotype *Tprn^+/+^* | Reference |  |  |  |  |
| *Tprn^+/-^* | 2.92 | [-5.91, 11.74] | 4.69 | 0.62 | 0.54 |
| *Tprn^-/-^* | 15.22 | [5.55, 24.86] | 5.13 | 2.97 | 3.9E-03** |
| Age P18 | Reference |  |  |  |  |
| P30 | 6.76 | [-6.58, 20.13] | 7.09 | 0.95 | 0.34 |
| P60 | 5.00 | [-5.02, 15.02] | 5.29 | 0.94 | 0.35 |
| Genotype x Age *Tprn^+/+^* P18 | Reference |  |  |  |  |
| *Tprn^+/-^* P30 | -1.26 | [-17.45, 15.01] | 8.60 | -0.15 | 0.88 |
| *Tprn^-/-^* P30 | 37.30 | [21.43, 53.08] | 8.39 | 4.45 | 3.1E-05*** |
| *Tprn^+/-^* P60 | -0.58 | [-12.35, 11.19] | 6.22 | -0.09 | 0.93 |
| *Tprn^-/-^* P60 | 55.00 | [42.18, 67.82] | 6.77 | 8.12 | 1.5E-10*** |
|  |  |  |  |  |  |
| **32 kHz** |  |  |  |  |  |
| (Intercept) | 22.86 | [13.50, 32.22] | 4.98 | 4.59 | 1.5E-05*** |
| Genotype *Tprn^+/+^* | Reference |  |  |  |  |
| *Tprn^+/-^* | 5.56 | [-5.39, 16.51] | 5.82 | 0.96 | 0.34 |
| *Tprn^-/-^* | 44.87 | [32.90, 56.84] | 6.37 | 7.05 | 4.9E-10*** |
| Age P18 | Reference |  |  |  |  |
| P30 | 5.48 | [-11.61, 22.57] | 9.08 | 0.60 | 0.55 |
| P60 | 12.14 | [-1.10, 25.38] | 7.04 | 1.73 | 0.09+ |
| Genotype x Age *Tprn^+/+^* P18 | Reference |  |  |  |  |
| *Tprn^+/-^* P30 | 1.94 | [-18.72, 22.59] | 10.98 | 0.18 | 0.86 |
| *Tprn^-/-^* P30 | 15.30 | [-4.93, 35.53] | 10.75 | 1.42 | 0.16 |
| *Tprn^+/-^* P60 | 13.32 | [-2.22, 28.87] | 8.26 | 1.61 | 0.11 |
| *Tprn^-/-^* P60 | 18.77 | [1.83, 35.70] | 9.00 | 2.08 | 0.04* |
