# Supplementary material for: Taperin bundles F-actin at stereocilia pivot points enabling optimal lifelong mechanosensitivity
Source: J Cell Biol. 2025 Jun 5;224(8):e202408026. doi: 10.1083/jcb.202408026 (PMC12139522; doi:10.1083/jcb.202408026)
Supplement: Table S5 — shows the comparisons of Tprn−/−, Tprn+/−, and Tprn+/+ ABR data between genotypes at each frequency and time point. [file jcb_202408026_tables5.docx]

Table S5. **Comparisons of *Tprn^-/-^*, *Tprn^+/-^*, and *Tprn^+/+^* ABR data between genotypes at each frequency and time point.**

| **8 kHz P18** | **Estimate** | ***s.e.*** | ***df*** | ***t* value** | ***p* value** |
| --- | --- | --- | --- | --- | --- |
| *Tprn^+/-^* - *Tprn^+/+^* | 0.98 | 5.16 | 83.00 | 0.19 | 0.98 |
| *Tprn^-/-^* - *Tprn^+/+^* | 14.81 | 5.65 | 83.00 | 2.62 | 0.03* |
| *Tprn^-/-^* - *Tprn^+/-^* | 13.83 | 4.43 | 83.00 | 3.12 | 6.9E-03** |
| **8 kHz P30** |  |  |  |  |  |
| *Tprn^+/-^* - *Tprn^+/+^* | 8.33 | 8.39 | 83.00 | 0.99 | 0.58 |
| *Tprn^-/-^* - *Tprn^+/+^* | 42.33 | 7.80 | 83.00 | 5.43 | 1.7E-06*** |
| *Tprn^-/-^* - *Tprn^+/-^* | 34.00 | 6.10 | 83.00 | 5.57 | 9.2E-07*** |
| **8 kHz P60** |  |  |  |  |  |
| *Tprn^+/-^* - *Tprn^+/+^* | 9.37 | 5.20 | 83.00 | 1.80 | 0.18 |
| *Tprn^-/-^* - *Tprn^+/+^* | 74.42 | 5.65 | 83.00 | 13.17 | 1.1E-10*** |
| *Tprn^-/-^* - *Tprn^+/-^* | 65.05 | 4.48 | 83.00 | 14.53 | 1.1E-10*** |
| **16 kHz P18** |  |  |  |  |  |
| *Tprn^+/-^* - *Tprn^+/+^* | 2.92 | 4.70 | 81.87 | 0.62 | 0.81 |
| *Tprn^-/-^* - *Tprn^+/+^* | 15.22 | 5.14 | 82.12 | 2.96 | 0.01* |
| *Tprn^-/-^* - *Tprn^+/-^* | 12.29 | 4.03 | 82.23 | 3.05 | 8.6E-03** |
| **16 kHz P30** |  |  |  |  |  |
| *Tprn^+/-^* - *Tprn^+/+^* | 1.66 | 7.56 | 82.76 | 0.22 | 0.97 |
| *Tprn^-/-^* - *Tprn^+/+^* | 52.52 | 7.04 | 82.86 | 7.46 | 3.3E-10*** |
| *Tprn^-/-^* - *Tprn^+/-^* | 50.86 | 5.52 | 82.98 | 9.22 | 1.1E-10*** |
| **16 kHz P60** |  |  |  |  |  |
| *Tprn^+/-^* - *Tprn^+/+^* | 2.34 | 4.74 | 81.9 | 0.49 | 0.87 |
| *Tprn^-/-^* - *Tprn^+/+^* | 70.22 | 5.14 | 82.1 | 13.65 | 7.0E-11*** |
| *Tprn^-/-^* - *Tprn^+/-^* | 67.88 | 4.07 | 82.3 | 16.66 | 7.8E-11*** |
| **32 kHz P18** |  |  |  |  |  |
| *Tprn^+/-^* - *Tprn^+/+^* | 5.56 | 5.83 | 83.00 | 0.95 | 0.61 |
| *Tprn^-/-^* - *Tprn^+/+^* | 44.87 | 6.38 | 83.00 | 7.03 | 1.7E-09*** |
| *Tprn^-/-^* - *Tprn^+/-^* | 39.31 | 5.01 | 83.00 | 7.85 | 1.5E-10*** |
| **32 kHz P30** |  |  |  |  |  |
| *Tprn^+/-^* - *Tprn^+/+^* | 7.50 | 9.48 | 83.00 | 0.79 | 0.71 |
| *Tprn^-/-^* - *Tprn^+/+^* | 60.17 | 8.82 | 83.00 | 6.82 | 4.1E-09*** |
| *Tprn^-/-^* - *Tprn^+/-^* | 52.67 | 6.90 | 83.00 | 7.63 | 2.1E-10*** |
| **32 kHz P60** |  |  |  |  |  |
| *Tprn^+/-^* - *Tprn^+/+^* | 18.89 | 5.88 | 83.00 | 3.21 | 5.2E-03** |
| *Tprn^-/-^* - *Tprn^+/+^* | 63.64 | 6.38 | 83.00 | 9.97 | 1.1E-10*** |
| *Tprn^-/-^* - *Tprn^+/-^* | 44.75 | 5.06 | 83.00 | 8.85 | 1.1E-10*** |
