# Supplementary material for: Taperin bundles F-actin at stereocilia pivot points enabling optimal lifelong mechanosensitivity
Source: J Cell Biol. 2025 Jun 5;224(8):e202408026. doi: 10.1083/jcb.202408026 (PMC12139522; doi:10.1083/jcb.202408026)
Supplement: Table S6 — shows the statistical analysis of ABR data from TprnN259/N259, Tprn+/N259, and Tprn+/+ mice. [file jcb_202408026_tables6.docx]

Table S6. **Statistical analysis of ABR data from *Tprn^N259/N259^*, *Tprn^+/N259^*, and *Tprn^+/+^* mice.**

|  | **Estimate** | **95% CI** | ***s.e.*** | ***t* value** | ***p* value** |
| --- | --- | --- | --- | --- | --- |
| (Intercept) | 15.89 | [7.28, 24.49] | 4.59 | 3.46 | 9.5E-04*** |
| Genotype *Tprn^+/+^* | Reference |  |  |  |  |
| *Tprn^+^*^/^*^N259^* | -5.36 | [-18.34, 7.97] | 6.90 | -0.78 | 0.44 |
| *Tprn^N259/N259^* | 40.73 | [30.66, 50.80] | 5.37 | 7.58 | 5.0E-10*** |
| Age P18 | Reference |  |  |  |  |
| P30 | 0.95 | [-9.52, 11.44] | 5.58 | 0.17 | 0.87 |
| P60 | 6.83 | [-4.49, 18.12] | 6.00 | 1.14 | 0.26 |
| Frequency 8 kHz | Reference |  |  |  |  |
| 16 kHz | 4.71 | [0.03, 9.40] | 2.44 | 1.93 | 0.06+ |
| 32 kHz | 17.98 | [13.30, 22.66] | 2.44 | 7.37 | 2.3E-11*** |
| Genotype x Age *Tprn^+/+^* P18 | Reference |  |  |  |  |
| *Tprn^+^*^/^*^N259^* P30 | 8.94 | [-6.63, 23.87] | 7.97 | 1.12 | 0.26 |
| *Tprn^N259/N259^* P30 | 0.88 | [-12.46, 14.23] | 7.09 | 0.12 | 0.90 |
| *Tprn^+^*^/^*^N259^* P60 | 10.99 | [-5.38, 26.71] | 8.40 | 1.31 | 0.19 |
| *Tprn^N259/N259^* P60 | 24.85 | [11.40, 38.60] | 7.17 | 3.46 | 7.3E-04*** |
| Observations: | 156 |  |  |  |  |
| Subjects: | 31 |  |  |  |  |
