# Supplementary material for: Taperin bundles F-actin at stereocilia pivot points enabling optimal lifelong mechanosensitivity
Source: J Cell Biol. 2025 Jun 5;224(8):e202408026. doi: 10.1083/jcb.202408026 (PMC12139522; doi:10.1083/jcb.202408026)
Supplement: Table S7 — shows the comparisons between time points of TprnN259/N259, Tprn+/N259, and Tprn+/+ ABR data within genotypes. [file jcb_202408026_tables7.docx]

Table S7. **Comparisons between time points of *Tprn^N259/N259^*, *Tprn^+/N259^*, and *Tprn^+/+^* ABR data within genotypes.**

| ***Tprn^+/+^*** | **Estimate** | ***s.e.*** | ***df*** | ***t* value** | ***p* value** |
| --- | --- | --- | --- | --- | --- |
| P30 - P18 | 0.95 | 5.66 | 79.41 | 0.17 | 0.98 |
| P60 - P18 | 6.83 | 6.10 | 97.77 | 1.12 | 0.50 |
| P60 - P30 | 5.89 | 4.88 | 134.43 | 1.21 | 0.45 |
| ***Tprn^+^*^/^*^N259^*** |  |  |  |  |  |
| P30 - P18 | 9.89 | 5.77 | 142.56 | 1.71 | 0.20 |
| P60 - P18 | 17.82 | 5.94 | 144.98 | 3.00 | 8.9E-03** |
| P60 - P30 | 7.93 | 4.39 | 132.52 | 1.81 | 0.17 |
| ***Tprn^N259/N259^*** |  |  |  |  |  |
| P30 - P18 | 1.83 | 4.44 | 130.32 | 0.41 | 0.91 |
| P60 - P18 | 31.69 | 3.95 | 137.22 | 8.02 | 1.3E-12*** |
| P60 - P30 | 29.86 | 4.76 | 143.54 | 6.28 | 1.1E-08*** |
