# Supplementary material for: Taperin bundles F-actin at stereocilia pivot points enabling optimal lifelong mechanosensitivity
Source: J Cell Biol. 2025 Jun 5;224(8):e202408026. doi: 10.1083/jcb.202408026 (PMC12139522; doi:10.1083/jcb.202408026)
Supplement: Table S8 — shows the statistical analysis of ABR data from Tprnin103/in103 and Tprn+/+ mice. [file jcb_202408026_tables8.docx]

Table S8. **Statistical analysis of ABR data from *Tprn^in103/in103^* and *Tprn^+/+^* mice.**

|  | **Estimate** | **95% CI** | ***s.e.*** | ***t* value** | ***p* value** |
| --- | --- | --- | --- | --- | --- |
| (Intercept) | 24.37 | [20.20, 28.54] | 2.17 | 11.22 | 3.8E-19*** |
| Genotype x Age P60 *Tprn^+/+^* | Reference |  |  |  |  |
| *Tprn^in103/in103^* P21 | 18.84 | [14.36, 23.33] | 2.34 | 8.06 | 1.8E-10*** |
| *Tprn^in103/in103^* P42 | 41.53 | [36.61, 46.44] | 2.56 | 16.21 | 4.2E-21*** |
| *Tprn^in103/in103^* P60 | 50.53 | [45.37, 55.68] | 2.69 | 18.80 | 8.8E-24*** |
| Frequency 250 Hz | Reference |  |  |  |  |
| 500 Hz | -22.69 | [-26.31, -19.08] | 1.86 | -12.23 | 2.3E-27*** |
| 1 kHz | -24.62 | [-28.23, -21.00] | 1.86 | -13.26 | 7.0E-31*** |
| 2 kHz | -19.81 | [-23.42, -16.19] | 1.86 | -10.67 | 3.2E-22*** |
| 4 kHz | -8.85 | [-12.46, -5.23] | 1.86 | -4.77 | 3.2E-06*** |
| 24 kHz | -2.12 | [-5.73, 1.50] | 1.86 | -1.14 | 0.26 |
| Observations: | 156 |  |  |  |  |
| Subjects: | 31 |  |  |  |  |
