# Supplementary material for: Taperin bundles F-actin at stereocilia pivot points enabling optimal lifelong mechanosensitivity
Source: J Cell Biol. 2025 Jun 5;224(8):e202408026. doi: 10.1083/jcb.202408026 (PMC12139522; doi:10.1083/jcb.202408026)
Supplement: Table S9 — shows the comparisons of ABR data from Tprnin103/in103 and Tprn+/+ mice at each frequency between genotype and age groups. [file jcb_202408026_tables9.docx]

Table S9. **Comparisons of ABR data from *Tprn^in103/in103^* and *Tprn^+/+^* mice at each frequency between genotype and age groups.**

| **4 kHz** | **Estimate** | **95% CI** | ***s.e.*** | ***t* value** | ***p* value** |
| --- | --- | --- | --- | --- | --- |
| [Intercept) | 56.67 | [51.58, 61.76] | 2.53 | 22.38 | 4.8E-27*** |
| Genotype x Age *Tprn^+/+^* P60 | Reference |  |  |  |  |
| *Tprn^in103/in103^* P21 | 13.33 | [6.76, 19.90] | 3.27 | 4.08 | 1.7E-04*** |
| *Tprn^in103/in103^* P42 | 30.83 | [23.63, 38.03] | 3.58 | 8.61 | 2.7E-11*** |
| *Tprn^in103/in103^* P60 | 33.33 | [25.78, 40.88] | 3.76 | 8.88 | 1.1E-11*** |
| **8 kHz** |  |  |  |  |  |
| (Intercept) | 30.83 | [24.58, 37.09] | 3.11 | 9.91 | 3.4E-13*** |
| Genotype x Age *Tprn^+/+^* P60 | Reference |  |  |  |  |
| *Tprn^in103/in103^* P21 | 8.61 | [0.54, 16.68] | 4.01 | 2.14 | 0.04* |
| *Tprn^in103/in103^* P42 | 36.67 | [27.82, 45.51] | 4.40 | 8.34 | 6.9E-11*** |
| *Tprn^in103/in103^* P60 | 51.17 | [41.89, 60.44] | 4.61 | 11.09 | 7.6E-15*** |
| **12 kHz** |  |  |  |  |  |
| (Intercept) | 22.50 | [17.52, 27.48] | 2.47 | 9.09 | 5.2E-12*** |
| Genotype x Age *Tprn^+/+^* P60 | Reference |  |  |  |  |
| *Tprn^in103/in103^* P21 | 19.17 | [12.74, 25.59] | 3.19 | 6.00 | 2.5E-07*** |
| *Tprn^in103/in103^* P42 | 41.67 | [34.63, 48.70] | 3.50 | 11.91 | 6.2E-16*** |
| *Tprn^in103/in103^* P60 | 59.50 | [52.12, 66.88] | 3.67 | 16.21 | 4.2E-21*** |
| **16 kHz** |  |  |  |  |  |
| (Intercept) | 24.17 | [18.68, 29.65] | 2.73 | 8.86 | 1.1E-11*** |
| Genotype x Age *Tprn^+/+^* P60 | Reference |  |  |  |  |
| *Tprn^in103/in103^* P21 | 20.83 | [13.75, 27.91] | 3.52 | 5.92 | 3.3E-07*** |
| *Tprn^in103/in103^* P42 | 51.67 | [43.91, 59.42] | 3.86 | 13.40 | 7.8E-18*** |
| *Tprn^in103/in103^* P60 | 60.83 | [52.70, 68.97] | 4.04 | 15.04 | 8.6E-20*** |
| **20 kHz** |  |  |  |  |  |
| (Intercept) | 33.33 | [26.92, 39.75] | 3.19 | 10.44 | 6.0E-14*** |
| Genotype x Age *Tprn^+/+^* P60 | Reference |  |  |  |  |
| *Tprn^in103/in103^* P21 | 28.89 | [20.60, 37.17] | 4.12 | 7.01 | 7.1E-09*** |
| *Tprn^in103/in103^* P42 | 50.83 | [41.76, 59.91] | 4.51 | 11.26 | 4.5E-15*** |
| *Tprn^in103/in103^* P60 | 56.67 | [47.15, 66.19] | 4.73 | 11.97 | 5.1E-16*** |
| **24 kHz** |  |  |  |  |  |
| (Intercept) | 48.33 | [42.56, 54.11] | 2.87 | 16.83 | 9.2E-22*** |
| Genotype x Age *Tprn^+/+^* P60 | Reference |  |  |  |  |
| *Tprn^in103/in103^* P21 | 22.22 | [14.77, 29.68] | 3.71 | 5.99 | 2.6E-07*** |
| *Tprn^in103/in103^* P42 | 37.50 | [29.33, 45.67] | 4.06 | 9.23 | 3.3E-12*** |
| *Tprn^in103/in103^* P60 | 41.67 | [33.10, 50.23] | 4.26 | 9.78 | 5.2E-13*** |
