# Supplementary material for: Taperin bundles F-actin at stereocilia pivot points enabling optimal lifelong mechanosensitivity
Source: J Cell Biol. 2025 Jun 5;224(8):e202408026. doi: 10.1083/jcb.202408026 (PMC12139522; doi:10.1083/jcb.202408026)
Supplement: Table S10 — shows the statistical analyses of Tprn−/−, Tprn+/−, and Tprn+/+ VsEP threshold, P1 amplitude, and P1 latency measures for each genotype and age. [file jcb_202408026_tables10.docx]

Table S10. **Statistical analyses of *Tprn^-/-^*, *Tprn^+/-^*, and *Tprn^+/+^* vestibular evoked potential (VsEP) threshold, P1 amplitude, and P1 latency measures for each genotype and age.**

| **VsEP** | **Estimate** | **95% CI** | ***s.e.*** | ***t* value** | ***p* value** |
| --- | --- | --- | --- | --- | --- |
| (Intercept) | -9.50 | [-11.10, -7.90] | 0.85 | -11.24 | 1.7E-16*** |
| Genotype *Tprn^+/+^* | Reference |  |  |  |  |
| *Tprn^+/-^* | -0.14 | [-2.06, 1.77] | 1.01 | -0.14 | 0.89 |
| *Tprn^-/-^* | 2.00 | [-0.54, 4.54] | 1.34 | 1.50 | 0.14 |
| Age P30 | Reference |  |  |  |  |
| P60 | -2.33 | [-4.60, -0.07] | 1.20 | -1.95 | 0.06+ |
| Genotype x Age *Tprn^+/+^* P30 | Reference |  |  |  |  |
| *Tprn^+/-^* P60 | 1.85 | [-0.92, 4.63] | 1.46 | 1.27 | 0.21 |
| *Tprn^-/-^* P60 | 1.33 | [-2.25, 4.92] | 1.89 | 0.71 | 0.48 |
|  |  |  |  |  |  |
| **P1 Amplitude** |  |  |  |  |  |
| (Intercept) | 1.51 | [1.15, 1.87] | 0.19 | 7.95 | 6.1E-11*** |
| Genotype *Tprn^+/+^* | Reference |  |  |  |  |
| *Tprn^+/-^* | -0.18 | [-0.61, 0.25] | 0.23 | -0.78 | 0.44 |
| *Tprn^-/-^* | -0.07 | [-0.62, 0.47] | 0.29 | -0.25 | 0.80 |
| Age P30 | Reference |  |  |  |  |
| P60 | -0.30 | [-0.78, 0.17] | 0.24 | -1.24 | 0.23 |
| Genotype x Age *Tprn^+/+^* P30 | Reference |  |  |  |  |
| *Tprn^+/-^* P60 | 0.36 | [-0.22, 0.94] | 0.30 | 1.21 | 0.24 |
| *Tprn^-/-^* P60 | -0.14 | [-0.90, 0.58] | 0.38 | -0.38 | 0.71 |
|  |  |  |  |  |  |
| **P1 Latency** |  |  |  |  |  |
| (Intercept) | 1.63 | [1.56, 1.69] | 0.03 | 47.30 | 2.3E-50*** |
| Genotype *Tprn^+/+^* | Reference |  |  |  |  |
| *Tprn^+/-^* | -0.03 | [-0.11, 0.04] | 0.04 | -0.83 | 0.41 |
| *Tprn^-/-^* | -0.01 | [-0.11, 0.08] | 0.05 | -0.29 | 0.78 |
| Age P30 | Reference |  |  |  |  |
| P60 | -0.01 | [-0.11, 0.08] | 0.05 | -0.27 | 0.79 |
| Genotype x Age *Tprn^+/+^* P30 | Reference |  |  |  |  |
| *Tprn^+/-^* P60 | 0.04 | [-0.07, 0.15] | 0.06 | 0.67 | 0.50 |
| *Tprn^-/-^* P60 | 0.00 | [-0.14, 0.14] | 0.08 | 0.01 | 0.99 |
| Observations: | 68 |  |  |  |  |
| Subjects: | 37 |  |  |  |  |
