# Supplementary material for: Taperin bundles F-actin at stereocilia pivot points enabling optimal lifelong mechanosensitivity
Source: J Cell Biol. 2025 Jun 5;224(8):e202408026. doi: 10.1083/jcb.202408026 (PMC12139522; doi:10.1083/jcb.202408026)
Supplement: Table S11 — shows the comparisons of Tprn−/−, Tprn+/−, and Tprn+/+ VsEP thresholds, P1 amplitude, and P1 latency between genotypes at specific ages. [file jcb_202408026_tables11.docx]

Table S11. **Comparisons of *Tprn^-/-^*, *Tprn^+/-^*, and *Tprn^+/+^* VsEP thresholds, P1 amplitude, and P1 latency between genotypes at specific ages.**

| **VsEP P30** | **Estimate** | ***s.e.*** | ***df*** | ***t* value** | ***p* value** |
| --- | --- | --- | --- | --- | --- |
| *Tprn^+/-^* - *Tprn^+/+^* | -0.14 | 1.01 | 61.00 | -0.14 | 0.99 |
| *Tprn^-/-^* - *Tprn^+/+^* | 2.00 | 1.34 | 61.00 | 1.50 | 0.30 |
| *Tprn^-/-^* - *Tprn^+/-^* | 2.14 | 1.17 | 61.00 | 1.83 | 0.17 |
|  |  |  |  |  |  |
| **VsEP P60** |  |  |  |  |  |
| *Tprn^+/-^* - *Tprn^+/+^* | 1.71 | 1.06 | 61.00 | 1.61 | 0.25 |
| *Tprn^-/-^* - *Tprn^+/+^* | 3.33 | 1.34 | 61.00 | 2.49 | 0.04* |
| *Tprn^-/-^* - *Tprn^+/-^* | 1.63 | 1.22 | 61.00 | 1.34 | 0.38 |
|  |  |  |  |  |  |
|  |  |  |  |  |  |
| **P1 Amplitude P30** |  |  |  |  |  |
| *Tprn^+/-^* - *Tprn^+/+^* | -0.18 | 0.23 | 60.51 | -0.78 | 0.72 |
| *Tprn^-/-^* - *Tprn^+/+^* | -0.07 | 0.29 | 60.51 | -0.25 | 0.97 |
| *Tprn^-/-^* - *Tprn^+/-^* | 0.10 | 0.25 | 60.51 | 0.42 | 0.91 |
|  |  |  |  |  |  |
| **P1 Amplitude P60** |  |  |  |  |  |
| *Tprn^+/-^* - *Tprn^+/+^* | 0.19 | 0.24 | 61.01 | 0.80 | 0.71 |
| *Tprn^-/-^* - *Tprn^+/+^* | -0.22 | 0.30 | 61.01 | -0.73 | 0.75 |
| *Tprn^-/-^* - *Tprn^+/-^* | -0.41 | 0.27 | 61.39 | -1.49 | 0.30 |
|  |  |  |  |  |  |
|  |  |  |  |  |  |
| **P1 Latency P30** |  |  |  |  |  |
| *Tprn^+/-^* - *Tprn^+/+^* | -0.03 | 0.04 | 62.00 | -0.83 | 0.69 |
| *Tprn^-/-^* - *Tprn^+/+^* | -0.01 | 0.05 | 62.00 | -0.29 | 0.96 |
| *Tprn^-/-^* - *Tprn^+/-^* | 0.02 | 0.05 | 62.00 | 0.42 | 0.91 |
|  |  |  |  |  |  |
| **P1 Latency P60** |  |  |  |  |  |
| *Tprn^+/-^* - *Tprn^+/+^* | 0.01 | 0.04 | 62.00 | 0.14 | 0.99 |
| *Tprn^-/-^* - *Tprn^+/+^* | -0.01 | 0.05 | 62.00 | -0.26 | 0.96 |
| *Tprn^-/-^* - *Tprn^+/-^* | -0.02 | 0.05 | 62.00 | -0.41 | 0.91 |
