# Supplementary figures and images for: Taperin bundles F-actin at stereocilia pivot points enabling optimal lifelong mechanosensitivity
Source: J Cell Biol. 2025 Jun 5;224(8):e202408026. doi: 10.1083/jcb.202408026 (PMC12139522; doi:10.1083/jcb.202408026)

Fig. 3 A      Fig. 3I

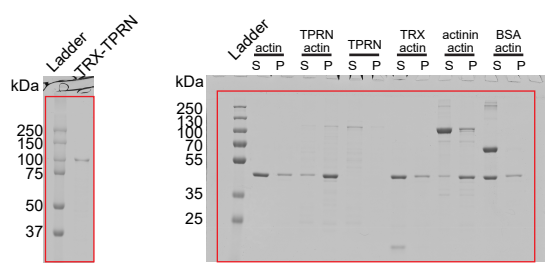

Fig. 3K

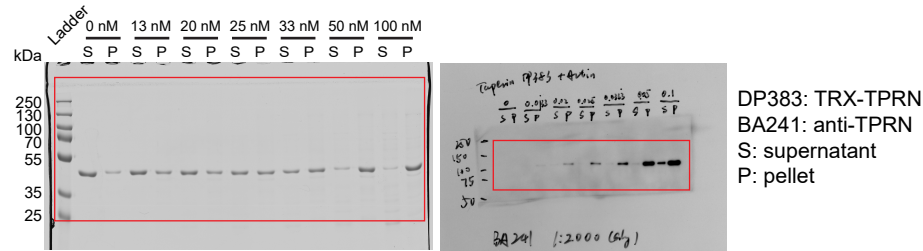

Supplement: SourceData F3 — is the source file for Fig. 3. [file jcb_202408026_sourcedataf3.pdf]

Fig. 5 B

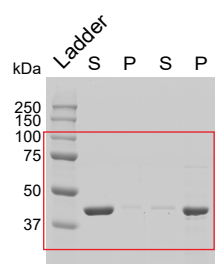

Fig. 5 C

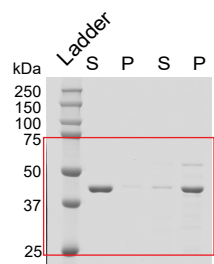

Fig. 5 D

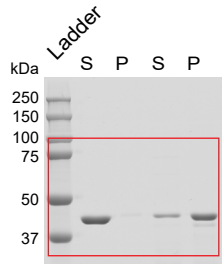

Fig. 5 E

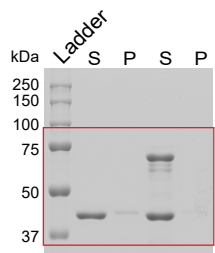

Fig. 5 F

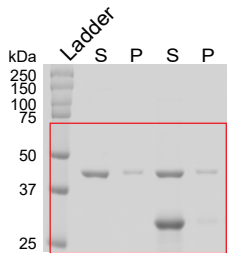

Supplement: SourceData F5 — is the source file for Fig. 5. [file jcb_202408026_sourcedataf5.pdf]

Fig. S1D

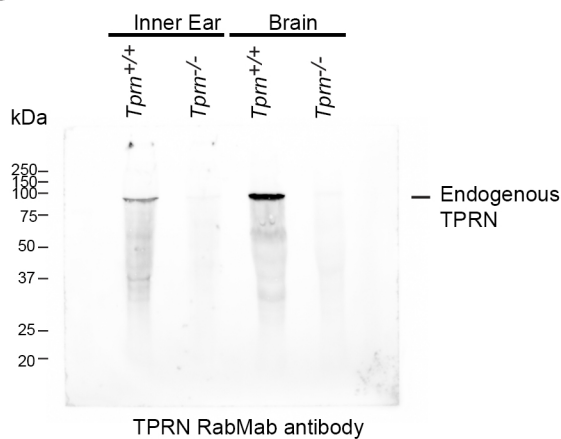

Fig. S1H

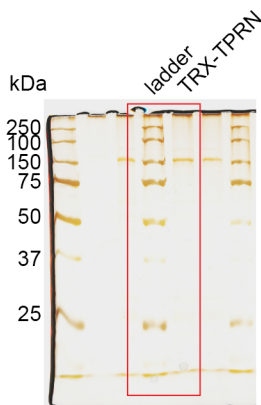

Fig. S1I

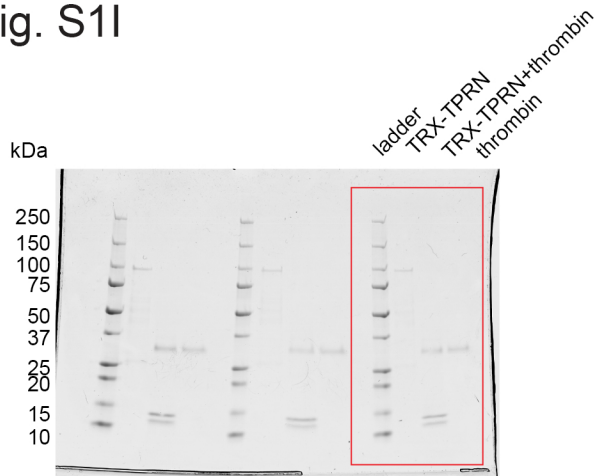

Fig. S1J

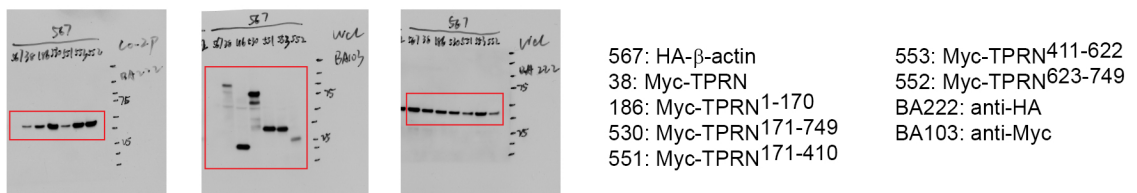

Fig. S1K

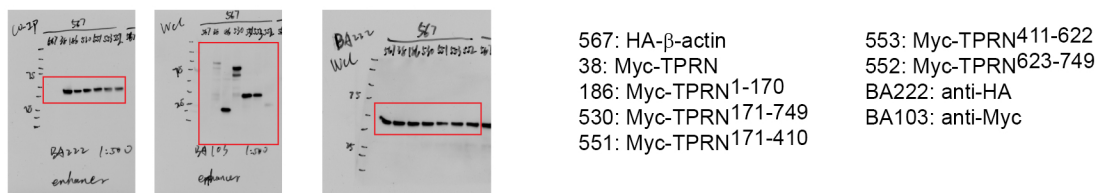

Fig. S1L

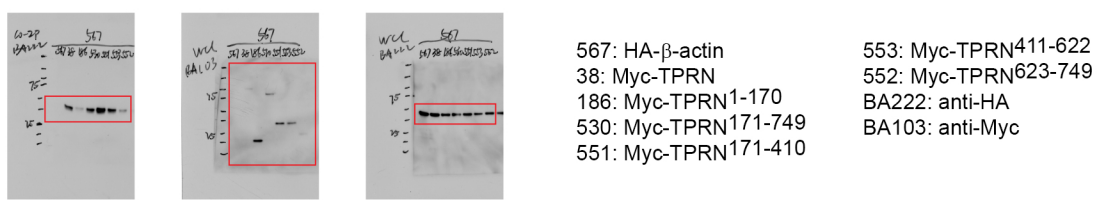

Supplement: SourceData FS1 — is the source file for Fig. S1. [file jcb_202408026_sourcedatafs1.pdf]
