# Supplementary material for: Taperin bundles F-actin at stereocilia pivot points enabling optimal lifelong mechanosensitivity
Source: J Cell Biol. 2025 Jun 5;224(8):e202408026. doi: 10.1083/jcb.202408026 (PMC12139522; doi:10.1083/jcb.202408026)

Fig. 4B

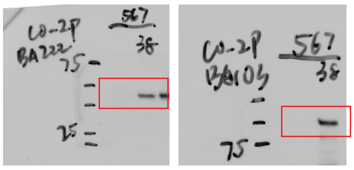

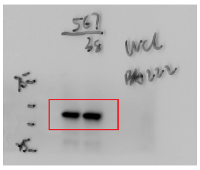

567: HA-β-actin
38: Myc-TPRN
BA222: anti-HA
BA103: anti-Myc

Fig. 4C

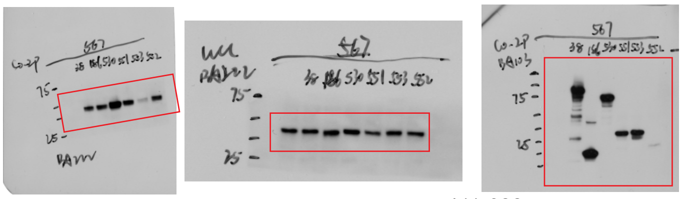

567: HA-β-actin
38: Myc-TPRN
186: Myc-TPRN$^{1-170}$
530: Myc-TPRN$^{171-749}$
551: Myc-TPRN$^{171-410}$
553: Myc-TPRN$^{411-622}$
552: Myc-TPRN$^{623-749}$
BA222: anti-HA
BA103: anti-Myc

Fig. 4D

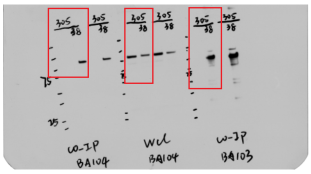

305: TPRN-EGFP
38: Myc-TPRN
BA104: anti-GFP
BA103: anti-Myc

Fig. 4E and 4F

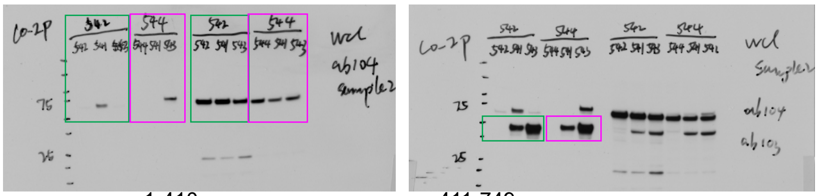

542: TPRN$^{1-410}$-EGFP
541: Myc-TPRN$^{1-410}$
543: Myc-TPRN$^{411-749}$

544: TPRN$^{411-749}$-EGFP
541: Myc-TPRN$^{1-410}$
543: Myc-TPRN$^{411-749}$

BA104: anti-GFP
BA103: anti-Myc

Fig. 4G

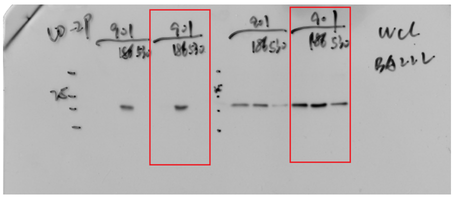

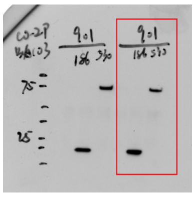

901: HA-TPRN$^{1-170}$
186: Myc-TPRN$^{1-170}$
530: Myc-TPRN$^{171-749}$
BA222: anti-HA
BA103: anti-Myc

Fig. 4H and 4I

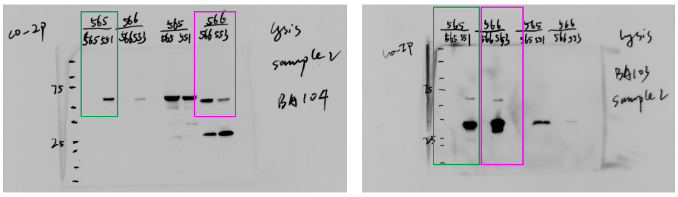

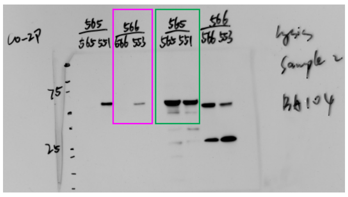

565: TPRN$^{171-410}$-EGFP
551: Myc-TPRN$^{171-410}$

566: TPRN$^{411-622}$-EGFP
553: Myc-TPRN$^{411-622}$

BA104: anti-GFP
BA103: anti-Myc

Fig. 4J

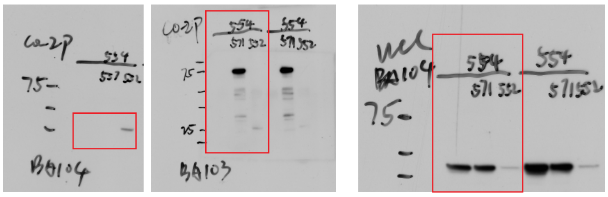

554: TPRN$^{623-749}$-EGFP
571: Myc-TPRN$^{1-622}$
552: Myc-TPRN$^{623-749}$
BA104: anti-GFP
BA103: anti-Myc

Note: Dual-color ladders (Bio-Rad) were used in the above Western blot. Due to space limitations, only the 75 kD and 25 kD bands were highlighted on the films. The other ladder bands were labeled on the film and their sizes are shown in the screenshot shown on the right (picture of ladder is downloaded from the Bio-Rad website).

Dual-color ladder

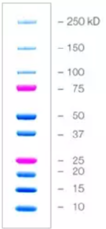

Supplement: SourceData F4 — is the source file for Fig. 4. [file jcb_202408026_sourcedataf4.pdf]
